# N-Acetylcysteine as a Host-Directed Therapy Against Clarithromycin-Resistant *Mycobacterium abscessus*

**DOI:** 10.3390/pathogens14040302

**Published:** 2025-03-21

**Authors:** Shuqi Yang, Ying Zhang, Jinchuan Xu, Zhenyan Chen, Yang Ren, Yujiao Long, Xuejiao Huang, Juanxi Liu, Huan Huang, Shiqi Xie, Ruiqing Ma, Yajuan Dong, Xiaoyong Fan, Zhidong Hu, Feng Li

**Affiliations:** 1 Shanghai Public Health Clinical Center, Fudan University, 2901 Cao Lang Road, Jinshan District, Shanghai 201508, China; 22211300006@m.fudan.edu.cn (S.Y.); zhangying6914@163.com (Y.Z.); jinchuanxu@hotmail.com (J.X.); 15680932726@163.com (Z.C.); renyang@shaphc.org (Y.R.); yjlong678@163.com (Y.L.); minhxj@163.com (X.H.); 23111010049@m.fudan.edu.cn (J.L.); 20110740035@fudan.edu.cn (H.H.); 23211300006@m.fudan.edu.cn (S.X.); rqma2014@126.com (R.M.); dongdong1141840250@163.com (Y.D.); xyfan008@fudan.edu.cn (X.F.); 2 Tuberculosis Research Center, Shanghai Public Health Clinical Center, 2901 Cao Lang Road, Jinshan District, Shanghai 201508, China; 3 Central Laboratory of Clinical Laboratory Diagnosis, Affiliated Hospital of Guizhou Medical University, Guiyang 550004, China; 4 Shanghai Institute of Infectious Diseases and Biosecurity, 138 Medical College Road, Xuhui District, Shanghai 200032, China

**Keywords:** *Mycobacterium abscessus*, N-acetylcysteine, non-tuberculous mycobacteria, clarithromycin, host-directed therapy, immune regulation

## Abstract

(1) Background: The treatment of *Mycobacterium abscessus* (*M. abscessus*) infections resistant to clarithromycin (CLR) is highly challenging. Traditional non-tuberculous mycobacteria (NTM) chemotherapy may disturb the immune homeostasis of the host by increasing oxidative stress; therefore, host-directed immunotherapy is an alternative option for infections caused by *M. abscessus*. (2) Method: A clinical isolate of CLR-resistant *M. abscessus* was screened, and then the therapeutic effects of N-acetylcysteine (NAC) against CLR-resistant *M. abscessus* infection were evaluated in Tohoku Hospital Pediatrics-1 (THP-1) cells and murine models. RNA sequencing and Western blot were used to profile the protective immune responses induced by NAC. The contribution of candidate signaling pathways was confirmed by the corresponding inhibitor and agonist. (3) Results: NAC immunotherapy led to a significant reduction in bacterial loads both in THP-1 cells and murine infection models, which was associated with enhanced antioxidant effects and downregulation of apoptosis signal-regulating kinase 1 (ASK1)–mitogen-activated protein ki-nase/extracellular signal-regulated kinase 3/6 (MKK3/6)–p38 mitogen-activated protein kinase (MAPK)-mediated inflammatory immune responses. The inhibitor of p38 signaling mimicked the protective effect of NAC, while the agonist attenuated it, suggesting that the p38 pathway is crucial in NAC-mediated immune protection against *M. abscessus* infection. (4) Conclusion: Our study suggests that NAC could be used as a host-directed therapy agent against drug-resistant *M. abscessus* infection.

## 1. Introduction

Non-tuberculous mycobacteria (NTM) are highly abundant in the environment, and epidemiological evidence suggests an overall increase in NTM infections in the past decade [1,2]. As an opportunistic environmental pathogen, *Mycobacterium abscessus* (*M. abscessus*) can cause progressive and fatal disease in susceptible patient populations [3,4,5] and is notorious for resistance to most classes of antibiotics, with a lengthy treatment duration and low success rates [6,7]. No antibiotic is formally approved for the treatment of *M. abscessus* disease, and *M. abscessus* exhibits resistance to the majority of medications used to treat tuberculosis [8,9]. Thus, the chemotherapeutic options for infections caused by *M. abscessus* are limited.

In the 1990s, clarithromycin (CLR) became the drug of choice for *M. abscessus* infection, with successful treatment [10]. Although a high level of resistance is observed, CLR and its related macrolide are still widely used today due, at least partially, to their immunomodulatory properties [6,11]. Thus, it has been proposed that immunomodulatory medications could be used on opportunistic infections such as *M. abscessus* by enhancing the host immune response [12,13]. However, T-cell exhaustion has been reported to impede the effectiveness of immunotherapy due to long-term chronic infections [14,15].

Host-directed immunotherapy targeting macrophages is regarded as another feasible option [16]. Upon infecting the respiratory tract, mycobacteria are first engulfed by alveolar macrophages through phagocytosis, and then cellular aggregates are formed to prevent the spread of infection [17,18]. This triggers a respiratory burst, resulting in the fast release of high levels of reactive oxygen species (ROS) [19,20]. However, the excessive generation of ROS can also harm host cells by intensifying inflammation, leading to oxidative stress that negatively affects immunological function [21]. N-acetylcysteine (NAC), a well-known ROS scavenger, has been used to treat several inflammatory diseases [21,22]. Particularly, studies have demonstrated that NAC can effectively exert anti-tuberculosis effects by regulating the host–pathogen redox balance [23,24].

Clinical studies have demonstrated the potential therapeutic value of NAC in improving lung function in adult patients with tuberculosis [24]. The sputum culture conversion rate was significantly higher in an NAC intervention group compared to a placebo group [25]. The pattern of proinflammatory response changed after NAC administration, suggesting that NAC treatment remodels the host immune response and that adjustment of the pro/anti-inflammatory balance is associated with standard anti-tuberculosis drugs that have synergistic effects [23]. Furthermore, it has been shown that NAC exerts a dual effect against *Mycobacterium avium* by inducing host β-defensin-2 expression [26]. Moreover, the mucolytic properties of NAC enhance the penetration of antibiotics into the bronchial mucosa, and its immunomodulatory effects may synergistically improve the host microenvironment [26]. Many studies have evaluated the antimicrobial potential of NAC in *Mycobacterium tuberculosis* and *Mycobacterium avium*, and most of the identified mechanisms can also be applied to *M. abscessus*, albeit with differences in the host-induced pathogenicity. Thus, NAC could be used as a host-directed immunotherapy to enhance the effectiveness of first- and second-line tuberculosis medications against drug-resistant *M. tuberculosis* [27,28]. However, the effect of NAC on *M. abscessus* infection has yet to be elucidated.

Herein, we evaluated NAC as a host-directed therapy against drug-resistant *M. abscessus* infection in Tohoku Hospital Pediatrics-1 (THP-1) cells and murine models. Our data showed that NAC immunotherapy led to a significant reduction in bacterial loads both in THP-1 cells and murine models. This reduction was associated with enhanced antioxidant effects and downregulation of the apoptosis signal-regulating kinase 1 (ASK1)–mitogen-activated protein kinase/extracellular signal-regulated kinase 3/6 (MKK3/6)–p38 mitogen-activated protein kinase (MAPK)-mediated inflammatory immune responses. Thus, this study suggests that NAC could be used as a host-directed therapy agent against drug-resistant *M. abscessus* infection.

## 2. Materials and Methods

### 2.1. Polymerase Chain Reaction (PCR) Sequencing

All clinical isolates of *M. abscessus* used in this study were acquired from the Clinical Laboratory Department of the Shanghai Public Health Clinical Center. DNA was recovered from the bacterial isolates and subjected to amplification using ERM_F (5′-TGCCCCGATATATCTTTGGAGC-3′) and ERM_R (5′-GATTCCACCGGTTAGGCCG-3′) primers. The resulting product was then analyzed using Snapgene (version: 7.2.1). The PCR experiments were conducted in a 50 µL solution, with cycling settings consisting of 30 cycles of 30 s at 95 °C, followed by 30 s at 60 °C and finally 60 s at 72 °C. The pre-denaturation step was carried out at 95 °C for a duration of 5 min. This was followed by 35 cycles of denaturation at 95 °C for 1 min, annealing at 64 °C for 1 min, and extension at 72 °C for 1.5 min. There was a final extension step at 72 °C for 10 min. These steps were performed using a kit from Vazyme (Nanjing, China). The PCR products were subjected to electrophoresis on a 1% agarose gel. The amplicons were dispatched for Sanger sequencing at Genewiz (South Plainfield, NJ, USA).

### 2.2. Drug Susceptibility Testing

NAC, CLR, Clofazimine (CFZ), Azithromycin (AZM), and Amikacin (AMK) (MCE, Shanghai, China) were dissolved in DMSO following the CLSI standard guidelines. The drugs were then serially double-diluted in triplicate using cation-adjusted Mueller Hinton (CAMHB) medium. The concentration range used was 16 mg/mL to 7.8125 µg/mL, 256 to 0.125 mg/L, 256 to 0.125 mg/L, and 256 to 0.125 mg/L. The concentration of *M. abscessus* was calibrated to 5 × 10^5^ Colony Forming Units (CFUs)/mL using the micro-broth dilution assay and introduced into round-bottom 96-well plates. The control group consisted of *M. abscessus* without any treatment, the negative group was composed of medium containing diluted DMSO, and the positive group was treated with rifampicin. The incubation process was carried out at 37 °C. To measure the Minimum inhibitory concentration (MIC) of CLR, incubation was necessary for a minimum of 7 days. For all other medicines, incubation was required for 3–5 days.

NAC sensitivity was assessed in conjunction with CLR and CFZ IN a round-bottom 96-well plate. CLR and CFZ were diluted in a serial manner down the horizontal axis, while NAC was diluted along the vertical axis. Similarly, the concentration of *M. abscessus* was adjusted to 5 × 10^5^ CFU/mL using drug-free medium as a growth control, with a final volume of 200 μL per well. The determination of the MICs of NAC and CLR in combination required an incubation period of 7 days or more at 37 °C, while all other medications required an incubation period of 3–5 days.

### 2.3. Bacterial and Cell Culture

*M. abscessus* was cultivated using Middlebrook 7H9 broth that was enriched with 0.5% glycerol, 0.05% Tween-80, and 10% oleic acid, dextrose, and catalase (OADC). THP-1 cells were obtained from ATCC and cultured in RPMI-1640 supplemented with 10% fetal bovine serum (FBS; Hyclone, Logan, UT, USA). To induce differentiation into M0 macrophages, the cells were stimulated with 100 nM Phorbol-12-myristate-13-acetate (PMA; MCE, Shanghai, China) for 48 h and then cultured in Dulbecco’s Modified Eagle Medium (DMEM) and incubated at 37 °C with 5% CO_2_.

### 2.4. Macrophage Intracellular Infection

As described previously [29], *M. abscessus* was treated as a single bacterium. The THP-1-derived M0 macrophages were infected with *M. abscessus* at a multiplicity of infection (MOI) of 1. After 4 h, the cells were rinsed three times to eliminate any *M. abscessus* outside the cells. Subsequently, NAC, a p38 agonist or inhibitor (MCE, Shanghai, China), was introduced to the cells and then treated for 24 h. The cells were disrupted, and 100 μL of the cell suspension was subjected to a 10-fold dilution gradient on Middlebrook 7H11 agar. After 4 days, the CFUs were counted.

### 2.5. Cell Viability Assay

Cytotoxicity was assessed using the Cell Counting Kit-8 (CCK-8) in 96-well plates containing 5 × 104 THP-1 cells per well. The cells were stimulated to differentiate into M0 macrophages, as previously described. Afterwards, the cells were exposed to NAC at various concentrations (5, 10, 20, 25, 30 mM) diluted in medium, along with a control group that received serum-containing media. The treatment lasted for 24 h in an incubator set at 37 °C and 5% CO_2_. A 10 μL volume of CCK-8 solution (MCE, Shanghai, China) was added to each well and incubated for an additional 2 h. The measurement of absorbance was conducted at an optical density of OD_600_ using an enzyme marker manufactured by Agilent BioTek (Santa Clara, CA, USA). Cell activity was standardized by subtracting the OD value of the blank control from the OD value of the experimental group.

### 2.6. Mice

Nude mice were purchased from specific pathogen-free (SPF) Biotechnology Co., Ltd. (Beijing, China). They were inoculated with 5 × 10^7^ CFUs of *M. abscessus* using a Glas-Col aerosol infection device. The bacterial quantities in the lungs were evaluated two hours after infection. On the third day after infection, NAC was administered through intraperitoneal (i.p.) injection at a dose of 150 mg/kg every other day. CLR and CFZ were administered orally once a day using a solution of carboxymethyl cellulose sodium at doses of 100 and 20 mg/kg, respectively. NAC was administered with a minimum interval of one hour from the other treatment medications to prevent any potential interaction. Seventeen days after being given the treatment, the mice were euthanized, and their bodies were placed on Middlebrook 7H11 agar and kept at 37 °C for 4 days. After that, the number of CFUs in the lungs and spleens of the infected mice was counted.

### 2.7. Pathology

Lung tissue slices underwent histological staining using Hematoxylin & Eosin (H&E) and antacid staining techniques. Microscopy (Olympus, Tokyo, Japan) was used to capture images of the pathological alterations. ImageJ software (version: 1.54d) was used to add scale bars, and inflammation was quantified in three fields of vision.

### 2.8. Cytometric Bead Array (CBA)

Mouse blood was obtained from the retro-orbital vein and allowed to rest at 4 °C for a duration of two hours. The blood was then subjected to centrifugation at a speed of 3000 rpm for 20 min at 4 °C. This process resulted in the separation of serum, which was subsequently tested for the presence of Interleukin-2 (IL-2), Interleukin-10 (IL-10), Interferon- gamma (IFN-γ), and Tumor Necrosis Factor-α (TNF-α) using the CBA assay, as per the guidelines provided by the manufacturer, and examined using flow cytometry (BD Biosciences, San Diego, CA, USA).

### 2.9. Oxidative Stress-Related Indicators

Assessment of total antioxidant capacity was conducted using a kit from Beyotime (Shanghai, China). The nitric oxide (NO) content was analyzed using the NO kit (Abbkine, Wuhan, China).

### 2.10. RNA Sequencing (RNA-Seq)

We obtained lungs from both the NAC-treated and control mice and then subjected them to transcriptome sequencing after 17 days of therapy. Total RNA was extracted from mouse lung tissue and transcriptome libraries were constructed using the VAHTS Universal V5 RNA-seq Library Prep kit according to the instructions. Transcriptome sequencing and subsequent analysis were performed by OE Biotech (Shanghai, China) using an Illumina NovaSeq 6000 system for sequencing RNA libraries. All raw RNA-seq data have been archived in SRA (PRJNA1236256).

### 2.11. Western Blot

Proteins were extracted from each group of mouse lung tissue homogenates using Radio Immunoprecipitation Assay (RIPA) lysis solution with protease inhibitors and phosphatase inhibitors (Epizyme Biotech, Shanghai, China). Protein concentration was determined using a Bicinchoninic Acid Assay (BCA) kit (Epizyme Biotech, Shanghai, China), and the proteins were denatured by boiling with loading buffer (Takara, Maebashi, Japan). The protein sample (10 μg) was separated using Sodium Dodecyl Sulfate–Polyacrylamide Gel Electrophoresis (SDS-PAGE) and subsequently deposited onto a 0.45 μm polyvinylidene fluoride (PVDF) membrane. The membrane was immersed in a solution of 5% skimmed milk for 1 h at room temperature. Following the addition of the primary antibody, the membrane was left to incubate overnight at 4 °C. It was then washed, and the secondary antibody (diluted at a ratio of 1:10,000) was added and incubated for 1 h at room temperature. Finally, the membrane was washed again. An enhanced chemiluminescence (ECL) solution was introduced and the bands were observed using chemiluminescence imaging equipment and measured using ImageJ.

The Western blot assay utilized the following primary antibodies: ASK1 (No: 28201-1-AP), p38 MAPK (No: 14064-1-AP), and p-p38 MAPK (No: 28796-1-AP) obtained from ProteinTech Group (Chicago, IL, USA). The p-MKK3/MKK6 (No: 12280) protein was obtained from Cell Signaling Technology (Danvers, MA, USA). The MEK3/MEK6 (No: A19830) protein was obtained from Abclonal (Wuhan, China). The p-ASK1 (No: 310216) protein was obtained from ZENBIO (Chengdu, China). The secondary antibody used was horseradish peroxidase (HRP)-conjugated goat anti-rabbit IgG (No: SA00001-2, 1:6000) from ProteinTech Group (Chicago, IL, USA).

### 2.12. Statistical Analysis

The data presented in this study are expressed as mean ± standard deviation. The results were analyzed using one-way analysis of variance. Additionally, *t*-tests were conducted to compare the NAC and control groups. The statistical analyses were conducted using GraphPad Prism software (version 9.5.0).

## 3. Results

### 3.1. Screening of Clinical Isolates of CLR-Resistant M. abscessus

Previous investigations showed that T/C polymorphisms at position 28 in the *M. abscessus erm(41)* gene are responsible for either inducible (T28) or intrinsic resistance (C28) to CLR [9]. In this study, eight clinical isolates of *M. abscessus* were collected; the MIC and *erm(41)* gene profiles are shown in Table 1. One highly CLR-resistant clinical isolate, Mab-4, which originated in a middle-aged male patient with few CD4^+^ T cells, was screened. Normally, *M. abscessus* exhibits resistance to CLR with an MIC ≥ 8 mg/L and to AMK with an MIC ≥ 64. In this study, the screened Mab-4 clinical strain exhibited strong resistance not only to CLR (MIC = 256 mg/L) but also to AMK (MIC = 64 mg/L) and AZM (MIC = 128 mg/L), as shown in Table 1 and Table 2. In addition, the MIC for NAC against Mab-4 was 16,000 mg/L, which is much higher than the physiological concentration in *M. abscessus*-infected patients [30], indicating that NAC cannot directly kill *M. abscessus* in vivo. NAC also exhibited a synergistic effect with both CFZ and CLR (Table 2), suggesting NAC would not inhibit their effects when used in combination.

### 3.2. NAC Exhibited Antimicrobial Activity Against Intracellular M. abscessus Infection

**Figure 1:**
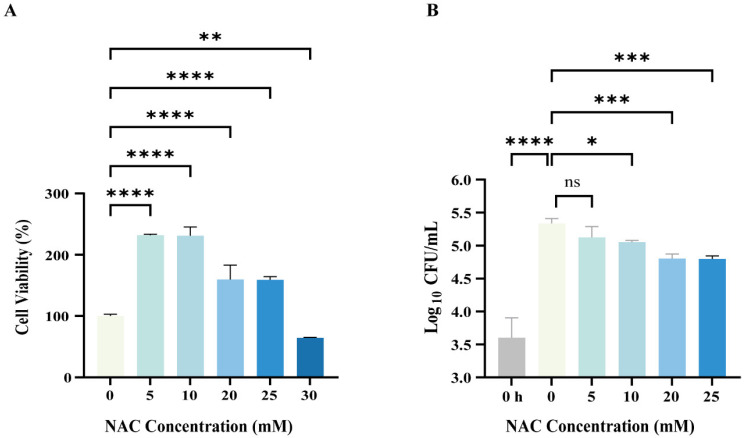
Intracellular bactericidal effects of *N-acetylcysteine (NAC)* on *Mycobacterium abscessus* (*M. abscessus)*-infected macrophages. (**A**) The M0 macrophages (Phorbol-12-myristate-13-acetate-stimulated Tohoku Hospital Pediatrics-1 cell lines) were treated with different concentrations of NAC for 24 h and tested by Cell Counting Kit-8 (CCK-8) assay; cell viability = [(NAC group-blank group)/(control group-blank group)] × 100%, *n* = 4. (**B**) Plot of intracellular bactericidal Colony Forming Units (CFUs) affecting M0 macrophages after 24 h of NAC treatment at different concentrations; 0 h represents the baseline CFUs of intracellular infection of macrophages after 4 h of *M. abscessus* infection, *n* = 4. The data are presented as means ± standard deviation. Statistical significance is indicated by asterisks: (ns, not significant; *, *p* < 0.05; **, *p* < 0.01; ***, *p* < 0.001; ****, *p* < 0.0001).

Macrophages are the initial and predominant phagocytes in the innate immune system. They can engulf *M. abscessus* within their cells and break them down by forming phagolysosomes. We first evaluated the effect of NAC on *M. abscessus*-infected macrophages. The administration of NAC did not exhibit cytotoxicity at concentrations ranging from 5 to 25 mM and even increased the cell viability of THP-1-derived M0 macrophages; however, this was not observed at a 30 mM concentration (Figure 1A). This also corroborates the previous finding that NAC has an extremely high in vitro MIC against CLR-resistant *M. abscessus* (Table 2) [31,32], suggesting that NAC controls the spread of *M. abscessus* not through a direct bactericidal effect but through its prominent protective properties against macrophages. *M. abscessus* infiltrates macrophages, where it continues to replicate and proliferate within the cell, ultimately rupturing the cell and disseminating it. For intracellular antimycobacterial effects, 20 mM and 25 mM NAC significantly inhibited the intracellular growth of *M. abscessus* by reducing ~0.5 log_10_ CFUs (Figure 1B). When NAC was utilized to clear *M. abscessus*, a dose-dependent bactericidal effect was observed with stepwise increases in concentration from 5 to 25 mM (Figure 1B). Thus, these data strongly suggest that NAC is highly effective in exerting intracellular antimicrobial activity against *M. abscessus*.

### 3.3. NAC Exhibited Anti-M. abscessus Activity in Murine Models

**Figure 2:**
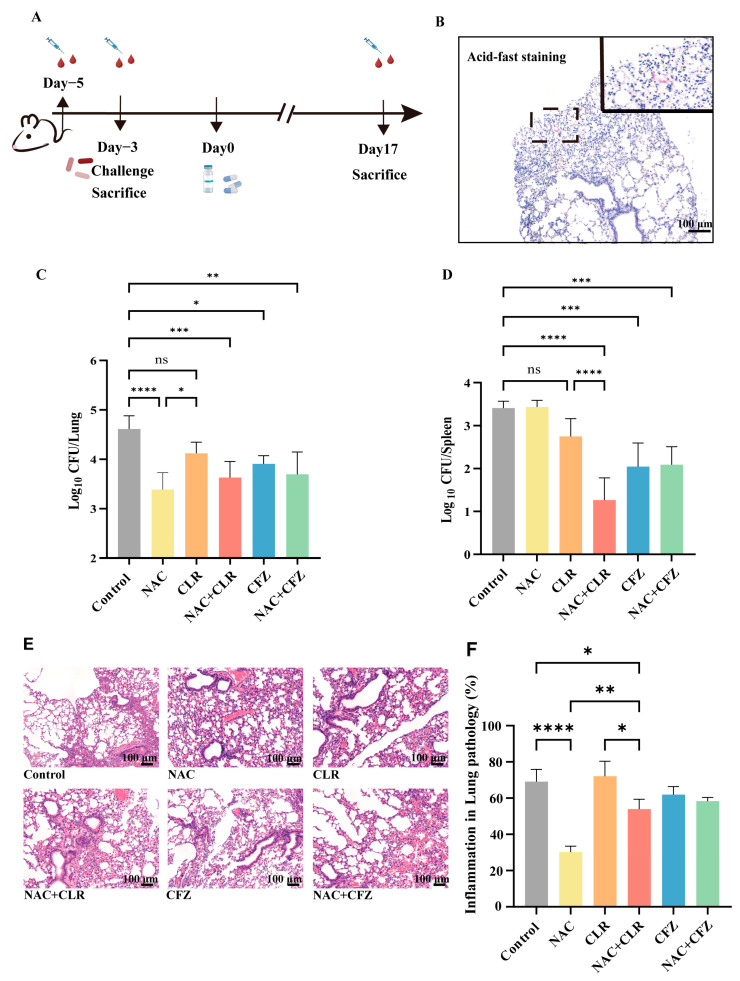
In vivo effects of NAC with CLR and CFZ on *M. abscessus*-infected nude mice. (**A**) A chronological record of the experiment involving the infection of nude mice. Five days prior to administration, nude mice were subjected to blood sampling from the retro-orbital vein. Two days later, they were exposed to *M. abscessus* via aerosolization for infection. Some mice were sacrificed to determine the CFUs, while blood sampling was also conducted. Administration of drugs (NAC, CLR, CFZ) commenced on the third day post infection. One week after infection, the CFUs of certain control mice were tallied. The CFUs of mice in each group were counted 17 days after medication administration. (**B**) A graph illustrating the staining of lung tissue sections from nude mice infected with *M. abscessus*, with a scale bar of 100 μm. Results of CFU counts in the lung tissues (**C**) and spleens (**D**) of nude mice; *n* = 5. The percentage of inflammation in lung histopathology sections stained with Hematoxylin & Eosin (H&E) (**E**) and semi-quantitative lung histopathology sections (**F**) was measured for each group. The scale bar measures 100 μm. The data are presented as means ± standard deviation. Statistical significance is indicated by asterisks: (ns, not significant; *, *p* < 0.05; **, *p* < 0.01; ***, *p* < 0.001; ****, *p* < 0.0001).

To evaluate the in vivo effect of NAC, nude mice were aerosol-infected with *M. abscessus* and treated with NAC (Figure 2A,B and Figure S1). The nude mice were used to eliminate the effect of T cells. Three weeks after infection, the mice were sacrificed. The data showed significantly lower bacterial loads in the lungs of the NAC group (Figure 2C) compared with the untreated group. To determine the effect of the drug in delaying the dissemination of *M. abscessus*, we assessed the change in bacterial load in the spleen and found no difference in the bacterial load in the spleen of the NAC and the untreated group (Figure 2D). The reduction in bacterial load was found to be most significant in the NAC combined with the CLR group, which was much better than in the NAC-treated group, probably due to the difference in the concentration of the drug reaching each organ. In addition, although no synergistic effect was observed in the lungs when combining NAC with CLR and CFZ (Figure 2C), the combination of NAC and CLR significantly reduced bacterial loads in the spleen (Figure 2D). The antimicrobial efficacy of NAC in combination with CFZ in the lungs was also superior to that of CFZ alone, suggesting that NAC may also have the potential to adjunctively augment some of the NTM therapeutic agents.

In addition, lung inflammation was assessed by H&E staining, and the data showed a significant reduction in the overall degree of inflammatory infiltration (Figure 2E) and lesion size (Figure 2F) in the NAC group compared with the control group. More importantly, the alveolar ratio tended to be normalized, and the alveoli were more structurally intact after NAC treatment (Figure 2E). Overall, the usage of NAC alone provided appreciable bactericidal effects in *M. abscessus*-infected pulmonary disease in a murine model.

### 3.4. NAC Reduced Proinflammatory Cytokines and Oxidative Stress Caused by M. abscessus Infection

**Figure 3:**
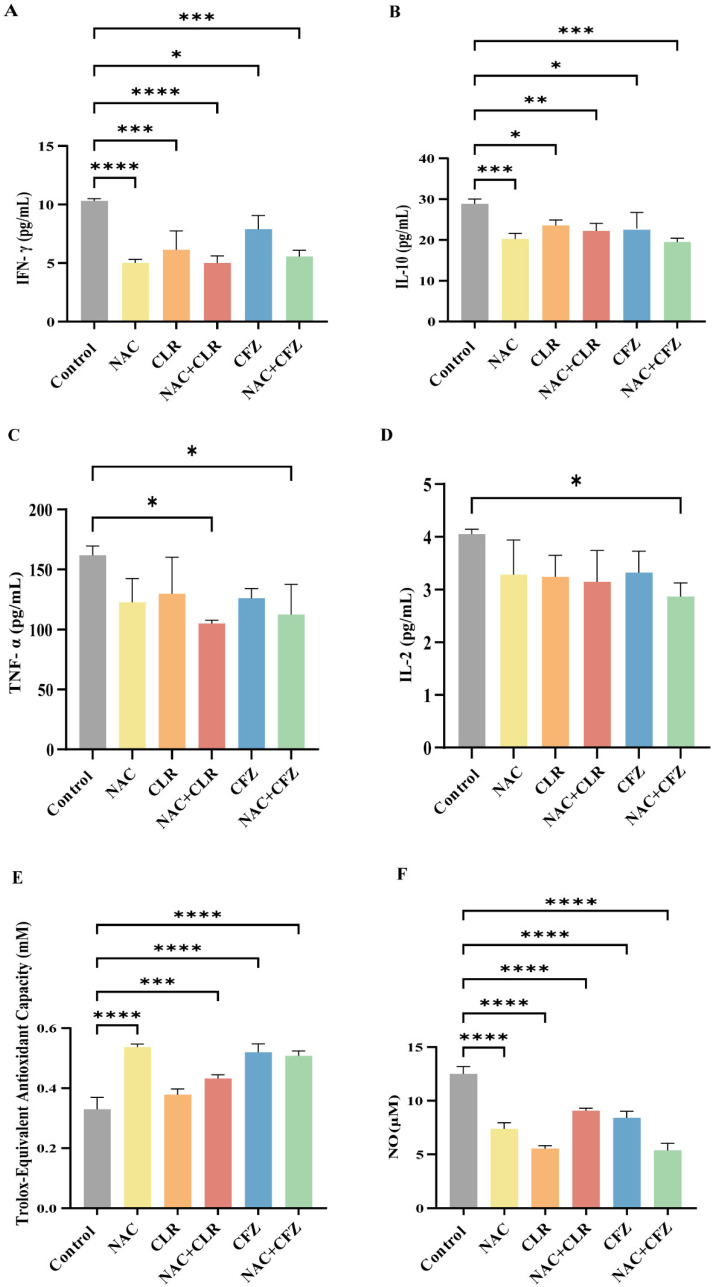
The inflammatory storm and oxidative stress in *M. abscessus*-infected mice treated with NAC combined with CLR and CFZ. (**A**–**D**) Plots displaying the levels of cytokines Interferon- gamma (IFN-γ) (**A**), Interleukin-10 (IL-10) (**B**), Tumor Necrosis Factor-α (TNF-α) (**C**), and Interleukin-2 (IL-2) (**D**) in the serum of infected nude mice after 17 days of treatment, *n* = 3. (**E**) A diagram illustrating the overall antioxidant capacity of nude mice following a 17-day therapy. (**F**) A graph depicting the concentrations of nitric oxide (NO) following a 17-day therapy in nude mice, *n* = 3. The data are presented as means ± standard deviation. Statistical significance is indicated by asterisks: (*, *p* < 0.05; **, *p* < 0.01; ***, *p* < 0.001; ****, *p* < 0.0001).

*M. abscessus* infection enhances the secretion of various proinflammatory cytokines, such as IFN-γ, TNF-α, and IL-2, which triggers cytotoxicity in infected macrophages, exerts antibacterial effects, and recruits and activates bystander cells. However, excessive systematic inflammation might disturb the homeostatic balance and result in tissue damage. In this study, we observed an overall suppression of proinflammatory cytokines (IFN-γ/TNF-α/IL-2; Figure 3A–C) and anti-inflammatory cytokines (IL-10) in the sera of infected nude mice in the treated groups compared with the control group (Figure 3A–D). In detail, NAC significantly decreased the expression levels of IFN-γ (Figure 3A) and IL-10 (Figure 3B) compared with the untreated group and modestly reduced the secretion of TNF-α (Figure 3C) and IL-2 (Figure 3D). IFN-γ is particularly recognized as a crucial controller of the inflammatory reaction caused by the invasion of the organism by *Mycobacterium*. *Mycobacterium* bacterial load is positively correlated with IFN-γ levels and corroborates the findings in Figure 2 [33]. The average levels of cytokines (IFN-γ, IL-10, TNF-α, IL-2) in infected nude mice exhibited a consistent decline compared to those in healthy mice (Figure S2). However, after NAC therapy, the cytokine levels typically increased and eventually reached a state similar to that of healthy mice (Figure S2). This implies that host-directed treatment by NAC restores cytokine fluctuations in post-infected mice.

Furthermore, we tested the antioxidant capacity, and the data showed that the NAC-treated group exhibited significantly higher levels than the control group (Figure 3E). Consistent with this observation, the release of NO was significantly lower in the NAC group (Figure 3F). These results indicate that NAC reduced proinflammatory cytokines by increasing the antioxidant capacity.

### 3.5. Suppression of MAPK-Related Pathways Following NAC Therapy in Mice Infected with M. abscessus

**Figure 4:**
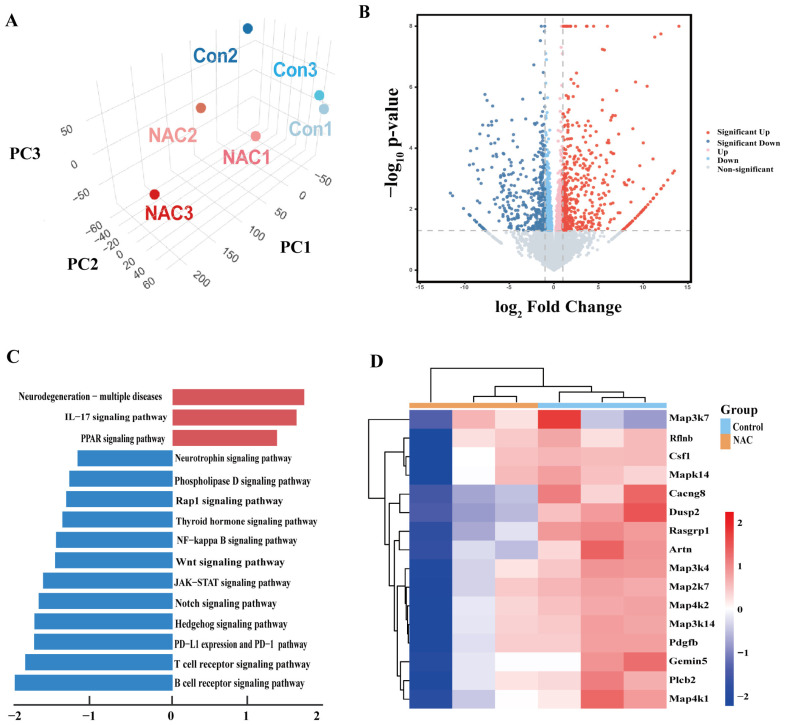
RNA sequence (RNA-seq) analysis of untreated and NAC-treated *M. abscessus*-infected mice. (**A**) A three-dimensional plot depicting the principal component analysis (PCA) of RNA-seq data from the control and NAC groups of the *M. abscessus* mice model. (**B**) A volcano plot comparing gene expressions between the NAC and control groups. (**C**) KEGG-based GSEA was performed on the differentially expressed genes in the NAC and control groups. The top 15 pathways were identified, and both up and downregulated pathways were ranked based on the gene ratio, which is the number of mapped genes divided by the number of genes in the pathway. (**D**) Heatmap displaying the expression levels of genes associated with the mitogen-activated protein kinase (MAPK) pathway in both the control and NAC groups.

To profile the genes and mechanisms affected by NAC, RNA-seq of lung tissue was conducted in this study. Principal component analysis (PCA) revealed distinctions between the NAC and control groups (Figure 4A). In comparison to the control group, the NAC group exhibited 878 genes that were differentially expressed (|Log10FC| > 1, FDR < 0.05). Among these genes, 498 were upregulated, and 380 were downregulated (Figure 4B). Kyoto Encyclopedia of Genes and Genomes (KEGG) pathway enrichment analysis revealed that the upregulated genes were significantly enriched in pathways related to Peroxisome proliferators-activated receptors (PPAR) signaling, and the downregulated genes were primarily enriched in Ras-related protein 1 (Rap-1), Nuclear factor kappa-light-chain-enhancer of activated B cells (NF-κB), and wingless-related integration (Wnt) signaling pathways (Figure 4C). A significant portion of the genes in these pathways were associated with p38 MAPK, in which the PPI networks of *Map3k4*, *Map2k7*, *Map2k6*, and *Mapk14* genes showed considerable overlap with p38 MAPK and its upstream regulators MKK3, MKK6, and ASK1 (Figure S3). Furthermore, these genes were shown to be downregulated in the NAC-treated group compared to the control group (Figure 4D).

ROS play a central role in the ASK1-MKK3/6-p38 signaling cascade by regulating cellular redox homeostasis. ROS deregulate the autoinhibitory state of ASK1 by directly oxidizing the Cys862/Cys719 residues of ASK1 and disrupting its complex with Trx [34]. ROS also decrease the activity of this kinase by inhibiting the Akt pathway and reducing the phosphorylation modification of the ASK1 Ser83 site by Akt [35,36,37]. Activated ASK1 acts similarly to MAP3K, phosphorylating conserved Ser/Thr residues of MKK3/6 and enhancing its kinase activity [34,38]. This process is positively regulated by ROS-mediated inactivation of protein tyrosine phosphatases [39]. MKK3/6 activates its catalytic function via dual phosphorylation of the Thr180/Tyr182 module of p38 MAPK [40]. Activated p38 MAPK, in turn, phosphorylates downstream transcription factors that drive pro-apoptotic gene expression, ultimately leading to apoptosis [41]. Intervention studies with the antioxidant NAC have provided new insights into the function of this pathway. NAC inhibits ASK1 activation by scavenging ROS and blocks the MKK3/6-p38 MAPK signaling cascade, thereby attenuating the inflammatory response and enhancing the bactericidal capacity of macrophages [42,43,44]. This finding suggests that targeting the ROS-ASK1-p38 axis may be a potential strategy for immunomodulation in NTM infection. Thus, we postulated that administering NAC to nude mice infected with *M. abscessus* might result in a decrease in the number of bacteria through suppression of the ROS-ASK1-MKK3/6-p38 MAPK axis.

### 3.6. The Antibacterial Effect of NAC Was Mediated by the p38 Signaling Pathway

**Figure 5:**
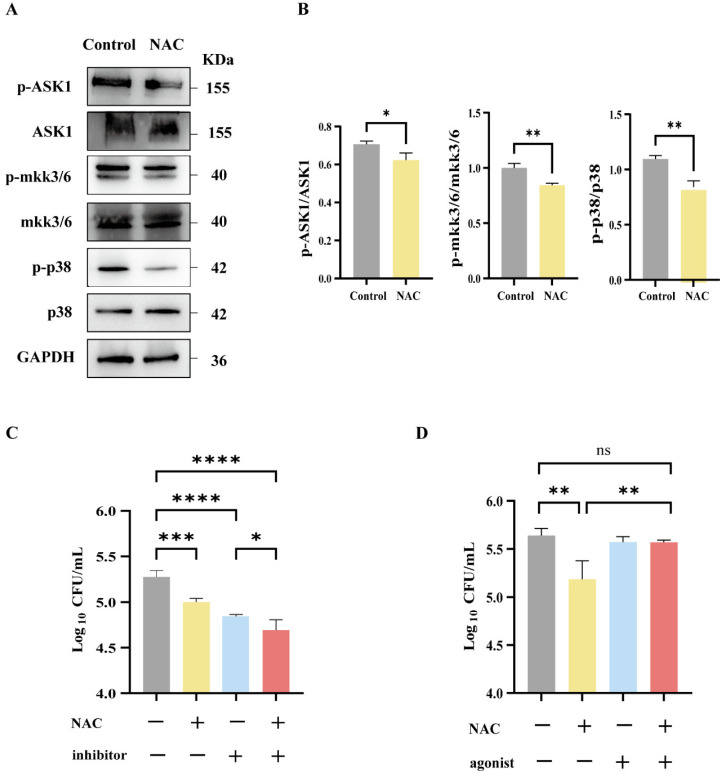
The antibacterial effect of NAC was mediated by the p38 MAPK signaling pathway. (**A**,**B**) Western blot strip plots (**A**) and semiquantitative plots (**B**) of lung tissue from nude mice 17 days after injection, *n* = 3. (**C**,**D**) Macrophages following infection with *M. abscessus*. Intracellular bactericidal CFU plots for 25 mM NAC and 10 μM p38 inhibitors (**C**) and 50 μM p38 agonists (**D**) in each group, *n* = 4. Statistical significance is indicated by asterisks: (ns, not significant; *, *p* < 0.05; **, *p* < 0.01; ***, *p* < 0.001; ****, *p* < 0.0001).

To further define the role of the ASK1-MKK3/6-p38 MAPK axis in NAC-mediated immune protection, we determined the expression levels of these proteins via Western blotting. Our data show that the phosphorylation levels of ASK1, MKK3/6, and p38 decreased in the NAC-treated group (Figure 5A,B). Further, the inhibitor of p38 was used to mimic the effect of NAC. As shown in Figure 5C, the CFU counts in the p38 inhibitor group were comparable with NAC treatment and were significantly lower than those of the untreated group, and their combination resulted in a greater antibacterial effect compared with either single treatment. By contrast, the p38 agonist significantly attenuated NAC-induced immune protection (Figure 5D). Thus, the NAC treatment suppressed the p38 signaling pathway, and p38 activation significantly attenuated NAC-induced immune protection against intracellular *M. abscessus* infection.

Taken together, during the process of *M. abscessus* infection, the release of excessive quantities of inflammatory cytokines by macrophages might result in tissue damage through the recruitment of other innate immune cells, such as neutrophils and natural killer cells, as well as the generation of ROS. Our data suggest that NAC could exhibit an antioxidant effect by downregulating the ASK1-MKK3/6-p38 MAPK axis, resulting in enhanced protective efficacy against *M. abscessus* infection.

## 4. Discussion

Current standard treatment protocols for *M. abscessus* infections have significant limitations; the oxidative stress microenvironment triggered by *M. abscessus* infections can activate ASK1 via oxidative modification, which then further phosphorylates MKK3/6, driving the dual phosphorylation of p38 MAPK. Notably, NAC intervention dose-dependently reduces ROS levels, and blocking this signaling cascade by maintaining ASK1 redox homeostasis provides a novel strategy for targeting the positive oxidative stress–inflammation feedback loop. Conventional drugs used to treat *M. abscessus* often have adverse effects that may lead to treatment interruption. NAC may mitigate the adverse effects of current anti-NTM drugs through its immunomodulatory effects. It is widely recognized that infection-induced oxidative stress may injure the host rather than act as a protective agent to kill the bacteria. As a result of excess ROS accumulation and cytokine secretion, p38 MAPK signaling will be upregulated. In principle, immunomodulators that are effective in reducing oxidative stress may protect macrophages against *M. abscessus*. Here, our study showed that NAC enhanced immune protection against *M. abscessus* infection through the inhibition of p38 signaling, which provides a candidate adjunctive host-directed therapy agent to fight *M. abscessus*.

Nude mice lacking T cells were chosen in our study for three reasons. Firstly, the abnormally elevated expression of the T-cell exhaustion markers Cytotoxic T lymphocyte-associated protein 4 (CTLA-4) and Programmed Cell Death Protein 1 (PD-1) suggests that a state of immunosuppression occurred in the microenvironment of the local infection site during NTM infection, where chronic infections such as *M. abscessus* might induce host T-cell depletion [34]. Thus, the T-cell deficiency characteristic of nude mice represents the state of T-cell depletion following chronic infection with *M. abscessus* in humans [45]. Secondly, the immunodeficient state of nude mice leads to a state of persistent chronic bacterial infection, which effectively mimics the inability to eradicate invading bacteria in patients with *M. abscessus* infections in the clinical setting. Excessive oxidative stress induced by *M. abscessus* infection not only produces direct cellular damage but also activates a proinflammatory immune response, which in turn triggers an excessive inflammatory response and ultimately leads to lung tissue damage. NAC has shown activity in a variety of potential therapeutic targeting pathways involving *Mycobacterium avium* infections, *Mycobacterium tuberculosis* infections, and others [35]. In this context, host-directed NAC (antioxidant) immunotherapy combined with chemotherapy might be more appropriate [36]. Finally, macrophages are the main colonizing cells of *M. abscessus*. Whereas our results showed that NAC can enhance the antimicrobial activity of macrophages (Figure 1), the T-cell-deficient model can be focused on to assess the direct modulatory effect of NAC on innate immune cells. Therefore, nude mice were selected in this study to simulate the state of T-cell functional failure in the late stage of infection and to exclude the interference of endogenous T cells in the assessment of NAC efficacy.

Given that NAC has been used as an expectorant in the treatment of chronic lung diseases, this offers translational potential for the clinical treatment of *M. abscessus* infections. Current studies still need to delve deeper into the specific role of MAPK in *M. abscessus* infections, as well as the mechanism of the ASK1-p38 pathway in the balance between host defense and immunopathology. In addition, exploring the clinical translational value of NAC or other antioxidants to improve the prognosis of *M. abscessus* infections by modulating this pathway will provide a theoretical basis for the further development of targeted therapies. Combining molecular biology and immunology tools to resolve the interaction network of the signaling pathway between *M. abscessus* and the host is a key direction for future research.

In this study, we found that NAC, in combination with anti-NTM drugs, significantly reduced bacterial load and improved the host inflammatory response, but its clinical translation still faces the challenge of side-effect management. Adjusting the chronokinetic dosing of NAC with anti-NTM drugs is critical for efficacy and toxicity. Although NAC has demonstrated unique advantages in enhancing the efficacy of anti-NTM drugs, its clinical dissemination needs to be combined with precise pharmacological monitoring and novel formulation technologies. In the future, multicenter clinical trials should be conducted to evaluate the benefit–risk ratio of NAC in drug-resistant strains and immunosuppressed populations and to establish real-time dose adjustment algorithms based on metabolomics. Future studies should focus on the development of individualized dosing algorithms, optimized dosing schedules, and innovative targeted delivery systems to ultimately achieve a precise balance between maximized efficacy and controllable toxicity.

The results of this study showed that NAC could be used as an immunomodulator to enhance the protection efficacy against *M. abscessus* infection, and they did not show negative effects when combined with other anti-NTM drugs. The mechanism analysis indicates that the downregulation of the ROS-ASK1-MKK3/6-p38 MAPK axis contributed to the enhanced protection, at least partially. Thus, our work strongly supports the potential of NAC as the primary therapeutic agent for *M. abscessus* rather than as an additional treatment option [46]. Further studies to conduct animal investigations for NAC in *M. abscessus* infection are warranted to determine the optimal blood concentration in patients before clinical application.

## 5. Limitations

Based on the limitations of current research models, nude mice were used in this study. Given the critical role of T cell-mediated adaptive immunity in host defense, in subsequent studies, an immunologically intact animal model should be used, and an *M. abscessus* infection model that is more closely related to clinical features should be constructed by introducing the phenotype of the underlying disease, for example, chronic lung disease. Thus, more murine models should be used before clinical application.

The effective in vitro concentration of NAC (20–25 mM) is significantly higher than the physiological level in humans. First, consider the time-compensation effect: in vitro experiments often feature short-term exposures (24–72 h) [47], while in vivo experiments feature continuous administration. It has been shown that treatment with 10 mM NAC for 6 h achieved an antioxidant effect equivalent to that achieved with 1 mM for 24 h. A similar antioxidant effect (achieved through the compensatory effect of the kinetics of glutathione synthesis, the precursor of NAC) can be achieved by short and long NAC treatments at high concentrations versus low concentrations.

First, the bioavailability limitations of NAC are taken into account: in vivo NAC undergoes a first-pass effect, resulting in low oral bioavailability, whereas in vitro experiments act directly on the cell with no metabolic loss. Second, in vivo, NAC needs to cross multiple barriers, such as the vascular endothelium and cell membrane, to reach the target, and its intracellular concentration is much lower than the plasma concentration. We will explore more NAC-matching therapeutic concentrations in vitro and in vivo for relevant experiments in future studies.

## Figures and Tables

**Table 1:** Information on patients, *M. abscessus* clinical isolates, the MIC of CLR, and the sequencing results of *erm(41)*.

				CLR MIC (mg/L)				
Patient	Strain	Source	Certain Disease	Day 3	Day 5	Day 7	Day 14	*erm(41)*
P-1	Mab-1	respiratory tract	AIDS	<0.25	<0.25	<0.25	<0.25	C28
P-2	Mab-2	respiratory tract	/	0.25	0.5	2	4	T28
P-3	Mab-3	respiratory tract	AIDS, DLBCL	0.5	1	2	8	T28
P-4	Mab-4	respiratory tract	AIDS	4	32	128	256	T28
P-5	Mab-5	respiratory tract	COPD	0.5	4	4	64	T28
P-6	Mab-6	respiratory tract	/	0.5	0.5	1	2	C28
P-7	Mab-7	cerebrospinal fluid	AIDS, syphilis	<0.25	<0.25	0.25	1	T28
P-8	Mab-8	respiratory tract	PTB, CHB	0.25	0.5	0.5	1	C28

Abbreviations: AIDS, acquired immunodeficiency syndrome; DLBCL, diffuse large B-cell lymphoma; COPD, chronic obstructive pulmonary disease; PTB, pulmonary tuberculosis; CHB, chronic hepatitis B.

**Table 2:** The MIC and FICI of Mab-4 on anti-NTM medicines.

Isolate	MIC (mg/L)				FICI	
	NAC	CFZ	AMK	AZM	NAC + CFZ	NAC + CLR
Mab-4	16000	8	64	128	0.3125	0.125

Abbreviations: FICI, fractional inhibitory concentration index; CLR, Clarithromycin; AMK, Amikacin; AZM, Azithromycin; CFZ, Clofazimine. FICI = (MIC of drug A in combination)/(MIC of drug A alone) + (MIC of drug B in combination)/(MIC of drug B alone). FICI values: synergy (FICI ≤ 0.5), indifference (0.5 < FICI ≤ 4.0), and antagonism (FICI > 4.0).

## Data Availability

All raw RNA-seq data have been archived in SRA (PRJNA1236256).

## Supplementary Materials

The following supporting information can be downloaded at https://www.mdpi.com/article/10.3390/pathogens14040302/s1, Figure S1: Bacterial load in the lungs of control nude mice; Figure S2: Trend plots showing serum cytokine levels (IFN-γ, IL-10, TNF-α, and IL-2) in nude mice infected with *M. abscessus*; Figure S3: A PPI network diagram illustrating the connections between ASK1, MKK3, MKK6, and p38 MAPK genes displayed using the Genemania database.
