# Supplementary material for: N-Acetylcysteine as a Host-Directed Therapy Against Clarithromycin-Resistant Mycobacterium abscessus
Source: Pathogens. 2025 Mar 21;14(4):302. doi: 10.3390/pathogens14040302 (PMC12030110; doi:10.3390/pathogens14040302)
Supplement: Supplementary file 1 [file pathogens-14-00302-s001.zip › pathogens-3525462-supplementary.pdf]

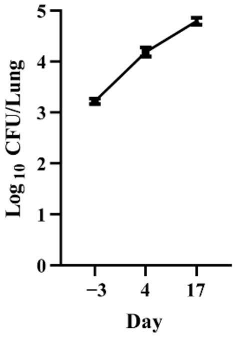

Figure S1. Bacterial load in the lungs of control nude mice. The bacterial quantities in the lungs of nude mice infected with *M. abscessus* were measured on the day of infection (3 days before treatment), on day 7 of infection (4 days after treatment), and on day 17 of treatment in the control group. The data presented are the mean values ± standard deviation of $\log_{10}$ CFU per lung, n=3.

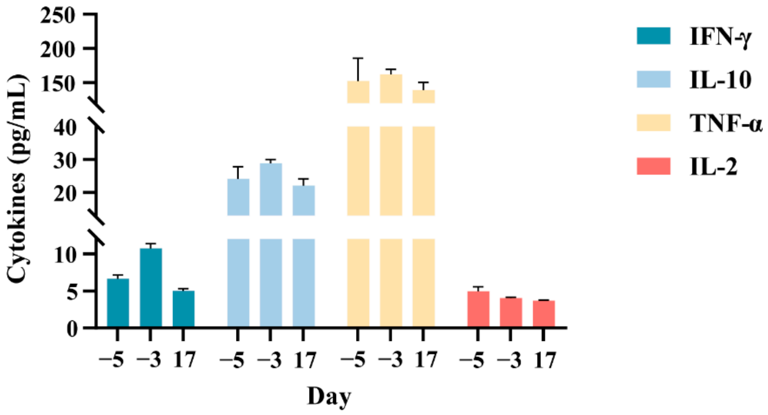

Figure S2. Trend plots showed serum cytokine levels in nude mice infected with *M. abscessus*, specifically for IFN-γ, IL-10, TNF-α, and IL-2. The graphs show the cytokine levels before infection, on the day of infection, and after 17 days of NAC treatment, n=3.

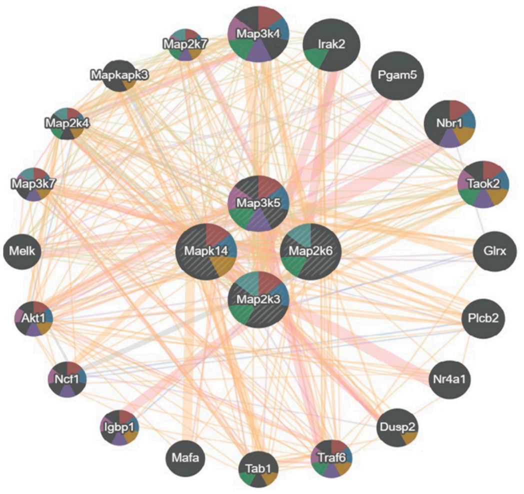

Figure S3. A PPI network diagram illustrating the connections between ASK1, MKK3, MKK6, and p38 MAPK genes is displayed using the Genemania database.
